# Supplementary material for: Sparse recurrent excitatory connectivity in the microcircuit of the adult mouse and human cortex
Source: eLife. 2018 Sep 26;7:e37349. doi: 10.7554/eLife.37349 (PMC6158007; doi:10.7554/eLife.37349)
Supplement: Supplementary file 1. [file elife-37349-supp1.docx]

**Features used in automated detection classifier**

| **Voltage Clamp** | **Current Clamp** |
| --- | --- |
| p-value of KS test comparing distributions of peak amplitude in the response window vs baseline window | p-value of KS test comparing distributions of peak deconvolved amplitude in the response window vs baseline window |
| p-value of KS test comparing the distribution of latency to peak in the response window vs the baseline window | p-value of KS test comparing the distribution of latency to peak in the response window vs the baseline window |
| Amplitude of fit to average of all responses | Amplitude of fit to average of all responses |
| Latency of fit to average of all responses | Latency of fit to average of all responses |
| Baseline potential of fit to average of all responses | Baseline potential of fit to average of all responses |
| Rise time of fit to average of all responses | Rise time of fit to average of all responses |
| Decay time constant of fit to average of all responses | Decay time constant of fit to average of all responses |
| Normalized RMS Error of fit to average of all responses | Normalized RMS Error of fit to average of all responses |
|  | Average of peak deconvolved amplitude of all responses |
|  | Average and standard deviation of latency to peak of all responses |

**Supplementary File 1.** Description of features extracted from raw data to use in synapse classifier
